# Challenges for clinical practice and research in family medicine in reducing the risk of chronic diseases. Notes on the EGPRN Spring Conference 2017 in Riga

**DOI:** 10.1080/13814788.2018.1429594

**Published:** 2018-02-02

**Authors:** Vija Silina, Ruth Kalda

**Affiliations:** ^a^ Department of Family Medicine, Riga Stradins University Riga Latvia; ^b^ Institute of Family Medicine and Public Health, University of Tartu Tartu Estonia

**Keywords:** Primary care, non-communicable diseases, prevention, early diagnosis, early treatment, multimorbidity

## Abstract

Chronic diseases in most cases belong to the category of non-communicable diseases (NCDs), which are the main cause of mortality globally. Cardiovascular diseases, diabetes, chronic obstructive pulmonary disease and cancer are the four NCDs responsible for 82% of NCD deaths. Prevention of NCDs implies health promotion activities that encourage healthy lifestyle and limit the initial onset of chronic diseases. Prevention also includes early detection activities, such as screening at-risk populations, as well as strategies for appropriate management of existing diseases and related complications. Early intervention, reducing morbidity and mortality rates could be an appealing idea for patients, physicians and governmental institutions but could also cause harm. Healthcare is undergoing profound changes, and the role of technology in diagnostics and management of chronic diseases in primary healthcare (PHC) is increasing remarkably. However, studies show that the standards of care for chronic diseases and preventive care are met by less than 50%. We still lack clear standards for patients with multiple chronic diseases. The applicability of a single evidence-based guideline to multimorbid patients is limited and can be problematic. Well-designed PHC studies focusing on the impact of medical interventions on morbidity, mortality and quality of life in the fields of early diagnosis, early treatment and multimorbidity are still needed.

KEY MESSAGES

- Use of early intervention to reduce morbidity and mortality rates of non-communicable diseases should be well justified to avoid harm.
- Educating both patients and clinicians on the harms and benefits of the early diagnosis interventions is essential.
- Care of multimorbid patients’ needs better designed PHC based research.

## Introduction

The theme of the EGPRN Riga Conference held in May 2017 was ‘Reducing the risk of chronic diseases in general practice/family medicine’. Chronic diseases in most cases belong to the category of non-communicable diseases (NCDs), which are the main cause of mortality globally. Cardiovascular diseases (CVDs), diabetes, chronic obstructive pulmonary disease and cancer are the four main NCDs, altogether responsible for 82% of NCD deaths.

**Figure 1.**
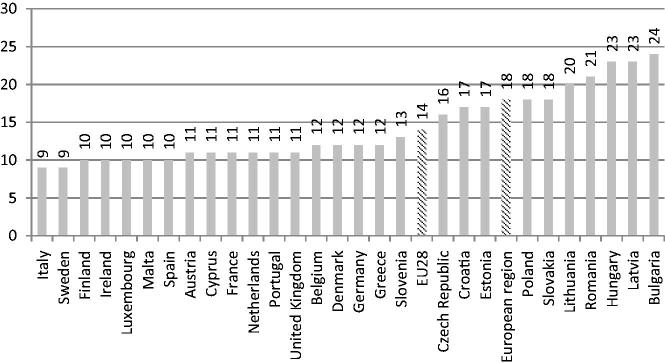
Probability (%) of premature death (dying between age 30 and 70 years) from four main NCDs (cardiovascular diseases, cancer, diabetes, chronic lung disease) in EU countries and in the WHO European region, data from 2015 [5].

Premature death is a major consideration when evaluating the impact of NCDs on a given population and is used as an indicator in the global monitoring framework. Approximately 42% of all NCD deaths globally occur before the age of 70 years (48% in low- and middle-income countries; 28% in high-income countries). Cardiovascular diseases are responsible for the largest proportion of NCD deaths under the age of 70 years (37%), followed by cancers (27%), chronic respiratory diseases (8%) and diabetes (4%) [1]. The probability of premature death (or probability of dying between the exact ages of 30 and 70 years) from the four main NCDs is used to assess the extent of the burden from mortality due to NCDs in the most economically productive population [2]. It can also be expressed as the percentage of 30-year-old people who would die before their 70th birthday from cardiovascular disease, cancer, diabetes or chronic respiratory disease, assuming that they would experience current mortality rates at every age and would not die from any other cause [3,4]. The probability of premature deaths from the four main NCDs has decreased from the year 2000 to 2015 both globally (from 22.7 to 18.8) and in the WHO European region (from 23.7 to 17.8). However, it varies a lot both among the WHO European region countries (from 8% in Iceland to 35% in Turkmenistan) and the European Union countries (from 9% in Italy and Sweden to 24% in Bulgaria), as shown in Figure 1 [5]. In 2013, approximately 2.5 million potentially productive life years were lost due to premature deaths from NCDs [6].

The prevention strategies for NCDs have been well described. A comprehensive global monitoring framework was adopted in 2013. It comprises nine targets and 25 indicators across three areas, with a focus on key outcomes, risk factors and national system responses to the challenge of NCDs [2,7]. There are four behavioural risk factors related to all four major NCDs: tobacco use; unhealthy diet (high sodium, saturated fat, trans-fat and sugar intake with low intake of fruits and vegetables); physical inactivity (less than 150 min of moderate intensity, or less than 75 min of vigorous intensity physical activity per week); and harmful use of alcohol [2,7,8]. The WHO European region has the highest prevalence of tobacco smoking among adults and the highest total per capita alcohol consumption among adults. The region shows a reduction in levels of physical activity across all age groups, has no countries meeting the WHO’s recommended level of 2 g sodium/day (equivalent to 5 g salt/day), and shows a steadily increasing prevalence of overweight and obesity [3]. Other targets of the framework include: relative reduction in risk of premature mortality from the four main NCDs; decreasing prevalence of raised blood pressure; stopping the rise in diabetes and obesity; implementing drug therapy and counselling to prevent heart attacks and strokes; improving availability of the affordable basic technology and essential medicines required to treat major NCDs [2].

Prevention is one of the primary healthcare (PHC) tasks well known to both physicians and public. Targets and quality criteria set for PHC teams, together with NCD-related morbidity and mortality data that is unfavourable, despite well identified primary and secondary prevention options [7], have encouraged the EGPRN that the conference in Riga was devoted to reducing the risk of chronic diseases in PHC.

This paper reflects on the ideas and research presented during the conference. First, we discuss some uncertainties in prevention and early diagnosis, including early cancer diagnosis and gut feelings. Later, the gaps in multimorbid patient care are addressed. Finally, we reflect on the potential use of general practitioners’ (GPs) routine data to promote realistic PHC-based studies.

## Prevention and early diagnosis

The idea of early intervention to outpace the emergence or progression of disease, and thus reduce morbidity and mortality rates, seems appealing to patients, physicians and governmental institutions. This idea is included in the European Definition of General Practice/Family Medicine. According to this document, the family physician ‘promotes health and well-being both by appropriate and effective intervention’ [9]. However, non-required interventions may cause harm, as well as waste valuable healthcare resources [9]. An abundance of studies supporting interventions, the trend to report positive findings and enthusiasm to apply the evidence of every positive effect, especially as promoted by secondary care specialists in their specific fields, can lead to a situation in which the remark about unnecessary interventions is neglected in real life. Physicians tend to overestimate the benefits and underestimate the harms of interventions [10]. Pressure from governmental institutions trying to fulfil recommendations set by international organizations (for example, NCD progress monitor indicators [11]) in primary care settings could add to this neglect. Besides, a wrong or delayed diagnosis has been mentioned as one of the most common errors in primary care [12,13]. The balancing of preventive and early diagnostic intervention with the patient’s safety and limited resources is a challenge in PHC. The best available external evidence supports national targets but the role of every individual GP and patient is sometimes neglected. Evidence-based medicine (EBM) has been defined as ‘conscientious, explicit, and judicious use of current best evidence in making decisions about the care of individual patients,’ and integrates the best external evidence with individual clinical expertise and the patients’ choices [14]. Respecting patients’ needs and expectations are particularly essential in primary care. As the majority of patients tend to overestimate intervention benefit and underestimate harm [15], it is crucial to educate patients on the harms and benefits of the interventions and to prepare them for inconclusive or unexpected test results to ensure that informed decisions are made [16].

## Early diagnosis of cancer

Cancer has been mentioned among the conditions most related to incorrect diagnosis [12]. Analyses of the results of widely implemented cancer screening programmes have led to an extensive discussion about overdiagnosis and overtreatment as threats to patient’s safety. Unfortunately, at the individual level, we can never be sure if the person is overdiagnosed [17]. This brings even more uncertainty to a family physician about what is right or wrong and how to detect cancer early only in those patients who would benefit from it.

Several studies and ideas related to the role of different factors in early diagnostics of cancer were presented at the EGPRN conference at Riga. Some countries, for example, Latvia, are still struggling with low cancer screening coverage rates. National screening programme response rates in Latvia do not exceed 50% for breast cancer and cervical cancer and 12% for colorectal cancer, despite the inclusion of these rates in the quality criteria for family physicians and the bonus payment for detecting first and second-stage cancer [18]. Analysing the reasons for the unwillingness of patients to participate (lack of time being one of the most often mentioned reasons), as well as implementing interventions in PHC could improve the situation, as presented by Ivanova et al. (Latvia) and Terjajeva et al. (Latvia) [19]. A review presented by Le Reste et al. (France) argues that colorectal cancer screening could be enhanced by training methods that modify physicians’ behaviour and train them to enhance patient-centred care communication instead of academic training methods [19].

Current knowledge on the potential harms of cancer screening programmes (particularly, false-positive results and overtreatment) combined with disproportionate resources needed to implement them should encourage more research and regular revision of such programmes. The focus should be on tests and interventions with the most favourable harm–benefit and cost-effectiveness ratios, as well as demand reasonable time resources from both medical staff and patients [16,17,20].

The Örenäs Research Group study presented by Harris M. [21] is working on the reasons for differences in relative cancer survival rates in European countries, which could be related to variations in how GPs act when faced with patients that could have cancer. This, in turn, is likely to be affected by how their healthcare systems are organized. The group has elaborated a validated questionnaire in 20 European languages to evaluate what system factors affect GPs’ decisions to refer patients for further investigation, how do these compare across different European countries, and how do they relate to one-year cancer survival rates. This study will supplement the knowledge presented in a study from the patient’s perspective in Denmark, which implies that the organizational structure of healthcare systems could influence care-seeking decisions, and that further primary care research is necessary [22].

## Gut feelings

To ensure that the right tests and procedures are applied to the right (highest risk) patients, it is necessary to understand what factors affect the family physicians’ clinical expertise or ability to select the right patients for diagnostic interventions. The ‘gut feeling’ could be described as intuitive fast thinking based on familiar patterns and context [23]. A family physician is an expert in recognizing patterns in their community and should be able to use effectively their gut feeling to select high and low-risk individuals. The presentation by Stolper et al. (Netherlands) indicated that gut feelings of patients are respected by PHC team members and they could influence a GP’s expertise and diagnostic reasoning [19]. However, external evidence on this issue still needs to be supplemented.

## Care of multimorbid patients

When applying external evidence to chronically ill patients with multimorbidity, the full definition of evidence-based medicine [14] should be particularly kept in mind. Besides, considering the differences in patient care among GPs at both national and international levels as demonstrated, for example, by Streit et al. [24], research in chronic disease management performed in the primary care setting is of utmost importance for the improvement of our knowledge and exchange of experience. The study implies that there is no real consensus on the clinical definition and management of frailty due to its complexity, which brings us back to the need for adequate teaching and research in this field.

The priorities for research include the general practitioners’ understanding of frailty, the problems with its care and cure as well as the understanding of the biological and psychosocial basis of frailty and evaluating the effects of therapeutic interventions.

Several presentations in the EGPRN conference at Riga were related to multimorbid patient care, searching for factors, which could affect the long-term care of chronically ill patients. Lalande et al. (France) [21] has found that age, frequency of family physician visits and the extent of family difficulties were the factors which predicted decompensation for multimorbid outpatients. Odorico et al., (France) [21] argues that the elderly in nursing homes decompensate more when they suffer from pain or when their entourage does not use coping strategies. Munoz et al. (Spain) [19] points out that non-adherence to the prescribed measures and respiratory infections were most related to heart failure decompensation in primary care settings.

Transfer of outer evidence to clinical practice is not so straightforward. We still lack clear standards for patients with multimorbidity. The applicability of a single evidence-based guideline to multimorbid patients is limited and can be problematic. A review including 10 studies shows that organizational interventions which targeted specific risk factor management or focused on areas where patients had more difficulties (such as medicines management), were more likely to be effective [25]. The NICE guideline from 2016 is the only one that covers the care of adults with multimorbidity. The guideline committee has made many recommendations for research, such as clinical- and cost-effectiveness of alternative approaches to organizing primary care compared with usual care for people with multimorbidity, as well as of stopping administering preventive medicines to people with multimorbidity who may not benefit from continuing them, etc. [26].

## Use of GPs’ routine data for research

Further research focusing on the impact of medical interventions on morbidity, mortality and quality of life in the fields of early diagnosis, early treatment and multimorbidity has to be encouraged. Reliable and complete data form an essential part of the study. Use of electronic health records (EHRs), including GPs’ routine data input in primary care settings, has broadened the research capacities, as the digitalization era allows EHRs to be combined to create much larger datasets than would be feasible manually, facilitating biomedical, epidemiological and public health research. However, it also presents certain challenges. The need to obtain informed consent could reduce the sample size and lead to selection bias [25]. Secure de-identifying of data for research purposes would solve the confidentiality issue [27]. Selection and matching of data from different databases in multicentre or international studies, including those that are not specially designed for research, is another challenge. Presentations by Mada et al. (Romania) [19] and Majnaric et al. (Croatia) [21] demonstrate the results of using EHRs’ data if they have not been prepared explicitly for research. Additionally, there is the challenge of de-identifying the data provided by GPs so that they could be used for diagnosis-specific studies both in primary and secondary care. Data could be particularly vulnerable in the case of rare diseases or if the location of the patient is known, as presented by Hauswaldt et al. (Germany) [19]. De-identification is also a challenge when data (for example, morbidity and mortality) are analysed in small populations or communities, which could be necessary if we would like to assess the probable impact of local environmental factors on health.

## Final remarks

Medicine and healthcare are undergoing profound changes. Technological innovation combined with automatization bring along exponential increase of data production. However, what we are going to do with the data? Can we synthesize useful information from the ‘big’ data, transfer it to clinical practice and put it into action when treating a single patient? Alternatively, can we just talk and listen to the patient, and try to understand the complexity and uniqueness of each patient? This is a million-dollar question, which deserves research.
